# Neural pathways for visual speech perception

**DOI:** 10.3389/fnins.2014.00386

**Published:** 2014-12-01

**Authors:** Lynne E. Bernstein, Einat Liebenthal

**Affiliations:** ^1^Department of Speech and Hearing Sciences, George Washington UniversityWashington, DC, USA; ^2^Department of Neurology, Medical College of WisconsinMilwaukee, WI, USA; ^3^Department of Psychiatry, Brigham and Women's HospitalBoston, MA, USA

**Keywords:** functional organization, audiovisual processing, speech perception, lipreading, visual processing

## Abstract

This paper examines the questions, what levels of speech can be perceived visually, and how is visual speech represented by the brain? Review of the literature leads to the conclusions that every level of psycholinguistic speech structure (i.e., phonetic features, phonemes, syllables, words, and prosody) can be perceived visually, although individuals differ in their abilities to do so; and that there are visual modality-specific representations of speech *qua* speech in higher-level vision brain areas. That is, the visual system represents the modal patterns of visual speech. The suggestion that the auditory speech pathway receives and represents visual speech is examined in light of neuroimaging evidence on the auditory speech pathways. We outline the generally agreed-upon organization of the visual ventral and dorsal pathways and examine several types of visual processing that might be related to speech through those pathways, specifically, face and body, orthography, and sign language processing. In this context, we examine the visual speech processing literature, which reveals widespread diverse patterns of activity in posterior temporal cortices in response to visual speech stimuli. We outline a model of the visual and auditory speech pathways and make several suggestions: (1) The visual perception of speech relies on visual pathway representations of speech *qua* speech. (2) A proposed site of these representations, the temporal visual speech area (TVSA) has been demonstrated in posterior temporal cortex, ventral and posterior to multisensory posterior superior temporal sulcus (pSTS). (3) Given that visual speech has dynamic and configural features, its representations in feedforward visual pathways are expected to integrate these features, possibly in TVSA.

## Introduction

This paper examines the questions, what levels of speech can be perceived visually, and how is visual speech represented by the brain? These questions would hardly have arisen 50 years ago. Mid-twentieth century speech perception theories were strongly influenced by the expectation that speech perception is an *auditory* function for processing *acoustic* speech stimuli (Klatt, 1979; Stevens, 1981), perhaps, in close coordination with the motor system (Liberman et al., 1967; Liberman, 1982). At the time, theorizing about speech perception was unrelated to evidence about visual speech perception (lipreading^1^), even though there were reports available in the literature showing that speech can be perceived visually. For example, there was extensive evidence during most of the twentieth century that lipreading can substitute for hearing in the education of deaf children (Jeffers and Barley, 1971), and there was evidence about the important role of lipreading in combination with residual hearing for children and adults with hearing impairments (Erber, 1971). The basic finding in normal-hearing adults that vision can compensate for hearing under noisy conditions was reported by mid-twentieth century (Sumby and Pollack, 1954). Even the report by McGurk and MacDonald (1976) that a visual speech stimulus mismatched with an auditory stimulus can alter perception of an auditory speech stimulus, an effect that has come to be known as the McGurk effect, had few responses in the literature until a number of years following its publication.

Research efforts to explain the McGurk effect and understand its general implications for speech perception and multisensory processing began in the 1980s (e.g., Massaro and Cohen, 1983; Liberman and Mattingly, 1985; Campbell et al., 1986; Green and Kuhl, 1989), as did forays into theoretical explanations for how auditory and visual speech information combines perceptually (Liberman and Mattingly, 1985; Massaro, 1987; Summerfield, 1987). In the following decade, in tandem with the development of new neuroimaging technologies, reports emerged that visual speech stimuli elicit auditory cortical responses (Sams et al., 1991; Calvert et al., 1997), results that seemed consistent with the phenomenal experience of the McGurk effect as a change in the auditory perception of speech. In the 1990s, breakthrough research on multisensory processing in cat superior colliculus was presented by Stein and Meredith (1993). Their evidence about multisensory neuronal integration provided a potential neural mechanism for explaining how auditory and visual speech information is processed (Calvert, 2001), specifically, that auditory and visual speech information converges early in the stream of processing.

Evidence for multisensory inputs to classically defined unisensory cortical areas (e.g., Falchier et al., 2002; Foxe et al., 2002) helped to shift the view of the sensory pathways as modality-specific until the levels of association cortex (Mesulam, 1998) toward the view that the brain is massively multisensory (Foxe and Schroeder, 2005; Ghazanfar and Schroeder, 2006). Findings suggesting the possibility that visual speech stimuli have special access to the early auditory speech processing pathway (Calvert et al., 1997; Ludman et al., 2000; Pekkola et al., 2005) were consistent with the emerging multisensory view. More recently, reconsideration of the motor theory of speech perception (Liberman and Mattingly, 1985) and mirror neuron system theory (Rizzolatti and Arbib, 1998; Rizzolatti and Craighero, 2004) have led inquiry into the role of somatomotor processing in speech perception, including visual speech perception (Hasson et al., 2007; Skipper et al., 2007a; Matchin et al., 2014). In this context, a question has been the extent to which visual speech is represented in frontal cortex (Callan et al., 2014). Thus, both the auditory and somatomotor systems have been studied for their roles in representing visual speech.

Curiously, the role of the visual system in representing speech has received less attention than the role of the auditory speech pathways. What is particularly curious is that the visual speech stimulus is psycholinguistically extremely rich, as shown below, yet there has been little research that has focused on how the visual system represents visible psycholinguistic structure (i.e., phonetic features, phonemes, syllables, prosody, and even words); although there have been, as we discuss below, multiple studies that show that speech activates areas in high-level visual pathways (for reviews, Campbell, 2008, 2011). The absence of pointed investigations of how visual speech is represented—in contrast to the detailed knowledge about auditory speech representations—is surprising, because sensory systems transduce specific types of energy such as light and sound, each affording its own form of evidence about the environment, including speech; and the current view of multisensory interactions does not overturn the classical hierarchical models of auditory and visual sensory pathways (e.g., Felleman and Van Essen, 1991; Kaas and Hackett, 2000; Rauschecker and Tian, 2000) as much as it enriches them. Clearly, the diverse evidence for multisensory interactions needs to be reconciled with evidence pointing to modality-specific stimulus representations and processing (Hertz and Amedi, 2014). This review explores the expectation that perception of visual speech stimuli requires visual representations of the stimuli through the visual pathways.

**Figure 1:**
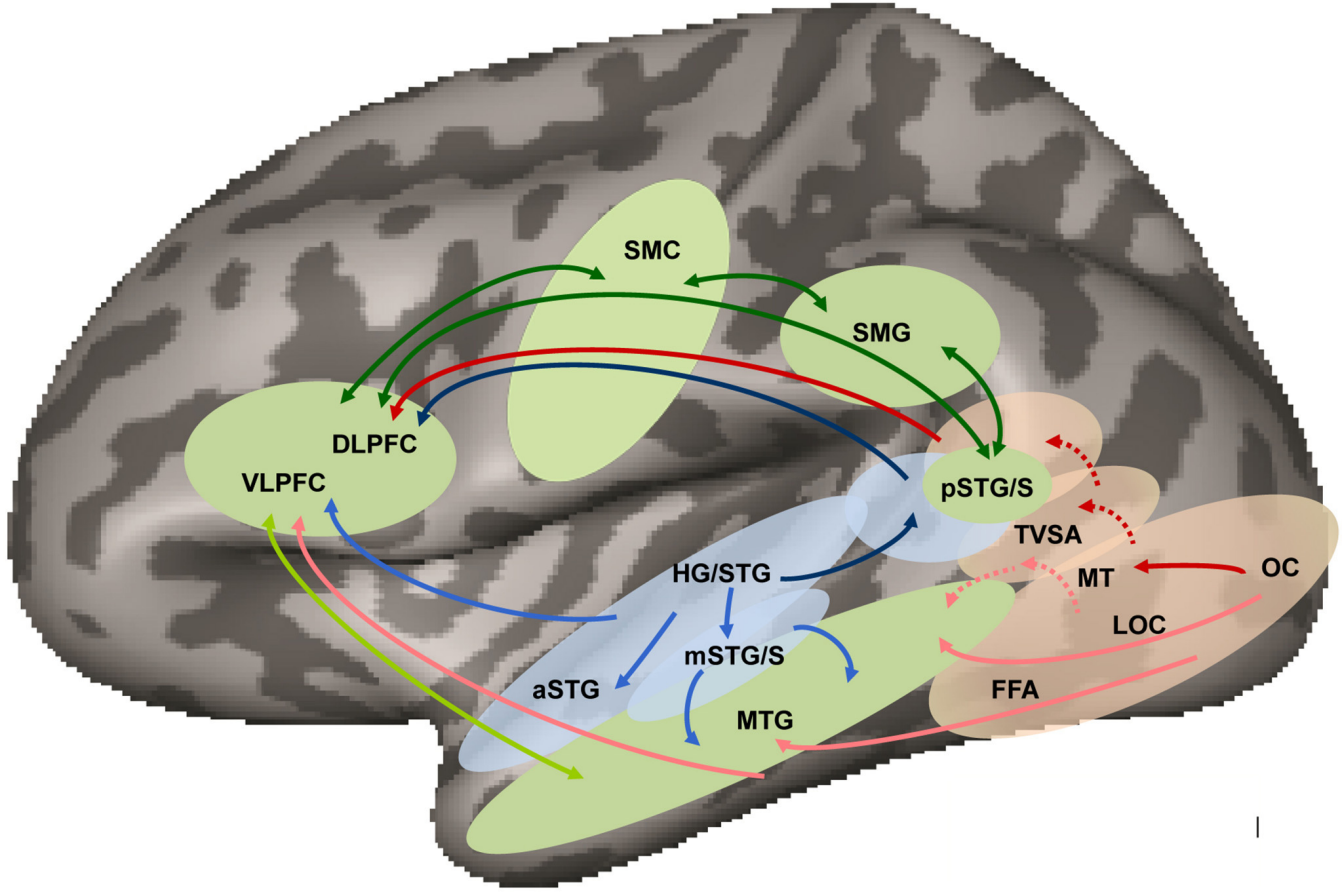
**Neuroanatomical working model of audiovisual speech perception in the left hemisphere based on models of dual visual (Wilson et al., 1993; Haxby et al., 1994; Ungerleider et al., 1998; Weiner and Grill-Spector, 2013) and auditory (Romanski et al., 1999; Hickok and Poeppel, 2007; Saur et al., 2008; Rauschecker and Scott, 2009; Liebenthal et al., 2010) pathways and audiovisual integration (Beauchamp et al., 2004) in humans**. Audiovisual speech is processed in auditory (blue) and visual (pink) areas projecting to amodal (green) middle temporal cortex via auditory (light blue arrows) and visual (light red arrows) ventral pathways terminating in VLPFC, and to multimodal posterior temporal cortex via auditory (dark blue) and visual (dark red) dorsal pathways terminating in DLPFC. Specialization for phoneme processing is suggested to exist in both auditory and visual pathways, at the level of mSTG/S and TVSA, respectively, although the pattern of connectivity of TVSA (shown in red dotted arrows), and whether it is part of the ventral and/or dorsal visual streams is unknown. Multimodal or amodal areas in the ventral and dorsal streams connect bi-directionally via direct and indirect ventral (light green arrows) and dorsal (dark green arrows) pathways. (HG/STG, Heschl's gyrus/superior temporal gyrus; aSTG, anterior superior temporal gyrus; mSTG/S, middle superior temporal gyrus and sulcus; pSTG/S, posterior superior temporal gyrus and sulcus; MTG, middle temporal gyrus; OC, occipital cortex; FFA, fusiform face area; LOC, lateral occipital complex; MT, middle temporal area; TVSA, temporal visual speech area; SMG, supramarginal gyrus; SMC, somatomotor cortex; VLPFC, ventrolateral prefrontal cortex; DLPFC, dorsolateral prefrontal cortex).

In this paper, we review the visual speech perception literature to support the view that every psycholinguistic level of speech organization is visible. That being the case, we consider the cortical representation of auditory speech as a possible model for the organization of visual speech processing. We suggest that research on the auditory organization of speech processing does not in fact encourage the notion that visual speech perception can be explained by multisensory connections alone. We propose a model that posits modality-specific as well as amodal speech processing pathways. Figure 1 summarizes our model, which is discussed in detail further below.

## Visual speech perception

### Implications of individual differences in lipreading ability

Any discussion of visual speech perception and its underlying neural mechanisms needs to acknowledge the fact of large inter-individual variation, both within and across normal-hearing and deaf populations (Bernstein et al., 2000, 2001; Auer and Bernstein, 2007; Tye-Murray et al., 2014). The differences are so large that findings on visual speech processing can probably not be accurately interpreted without knowing something about individual participants' lipreading ability and auditory experience.

For example, in a test of words correctly lipread in isolated sentences, the scores by deaf lipreaders ranged from zero to greater than 85% correct (Bernstein et al., 2000). Deaf lipreaders were able to identify as many as 42% of isolated monosyllabic words from a list of highly confusable rhyming words (each test word rhymed with five other English words). Among adults with normal hearing, there was a narrower performance range for the same stimulus materials: There were individuals with scores as low as zero and ones with very good lipreading ability with scores as high as 75% correct words in sentences and 24% correct on the isolated rhyming words. Analyses of phoneme confusions in lipreading sentences suggested that the deaf participants were using more visual phonetic feature information than the hearing adults. But the individual variation in lipreading sentences accounted for by isolated word vs. isolated phoneme identification (using non-sense syllables) scores showed that isolated words accounted for more variance than phonemes: Word identification scores with isolated rhyme words accounted for between 66 and 71% of the variance in words-in-sentences scores for deaf lipreaders and between 44 and 64% of the variance for normal-hearing lipreaders, values commensurate with other reports (Conklin, 1917; Utley, 1946; Lyxell et al., 1993). In Bernstein et al. (2000), phoneme identification in non-sense syllables accounted for between 21 and 43% of the variance in words-in-sentences scores for deaf lipreaders and between 6 and 18% of the variance for normal-hearing lipreaders. When regression was used to predict words-in-sentences scores, only participant group (deaf, normal-hearing) and isolated word scores were significant predictors (multiple *R* between 0.88 and 0.90). Additional studies confirm that the best lipreaders experienced profound congenital hearing loss, but that even among normal-hearing adults there are individuals with considerable lipreading expertise (Mohammed et al., 2006; Auer and Bernstein, 2007).

Individuals with hearing impairments may rely primarily on visual speech, even in the context of hearing aid and cochlear implant usage (Rouger et al., 2007; Bernstein et al., 2014; Bottari et al., 2014; Song et al., 2014). Lipreading ability in individuals with hearing loss, including those with congenital impairments is likely associated with a wide range of neuroplastic effects, including take-over of auditory processing areas by vision (Karns et al., 2012; Bottari et al., 2014) or somatosensation (Levanen et al., 1998; Auer et al., 2007; Karns et al., 2012), and alterations of sub-cortical connections (Lyness et al., 2014).

### Visible levels of speech

From a psycholinguistic perspective, speech has a hierarchical structure comprising features, phonemes, syllables, words, phrases, and larger units such as utterances, sentences, and discourse. The questions here are which of these levels can be perceived visually, and whether any type of these speech patterns is represented in visual modality-specific areas. As with auditory speech perception, we expect that at a minimum visual speech perception extends to the physical properties of speech, that is, its *phonetic* feature properties, and that those properties express the vowels, consonants, and prosody of a language. The term *phonemic* refers to language-specific segmental (vowel and consonant) properties. Thus, for example, the term *phonetic* applies to speech features without necessarily specifying a particular language, and *phonemic* refers to segmental distinctions used by a particular language to distinguish among words (Catford, 1977). Prosody comprises phonetic attributes that span words or phrases, such as lexical stress in English (e.g., the distinction between the verb in “to re*cord*” and the noun in “the *re*cord”), and intonation (e.g., pronunciation of the same phrase as an exclamation or a statement, “we won!/?”). Necessarily, physical acoustic phonetic speech signals are different than optical phonetic speech signals; and although they may convey the same linguistic content, they are expected to be represented initially by different peripheral, subcortical, and primary sensory areas that code different low-level basic sensory features (e.g., light intensities vs. sound intensities, spatio-temporal vs. temporal frequencies, etc.). As we suggest below, there is the possibility that modality-specific representations exist to the level of whole words. But we do not expect separate representations of the meanings of individual words or of whole visual multi-word utterances, although there may be highly frequent utterances that are represented as such.

### Features, phonemes, and visemes

Speech production simultaneously produces the sounds and sights of speech, but the vocal tract shapes, glottal vibrations, and velar gestures that produce acoustic speech (Stevens, 1998) are not all directly visible. Some of them are visible as correlated motions of the jaw and the cheeks (Yehia et al., 1998; Jiang et al., 2002, 2007). An ongoing idea in the literature is that visual speech is too impoverished to convey much phonetic information (Kuhl and Meltzoff, 1988). This idea is supported by examples of poor lipreading performance and by focusing on how acoustic signals are generated. For example, the voicing feature (i.e., the feature that distinguishes “b” from “p”) is typically expressed acoustically in pre-vocalic position in terms of glottal vibration characteristics such as onset time (Lisker et al., 1977). But the glottis is not a visible structure, so a possible inference is that the voicing feature cannot be perceived visually. However, there are other phonetic attributes that contribute to voicing distinctions. For example, post-vocalic consonant voicing depends greatly on vowel duration (Raphael, 1971), and vowel duration—the duration of the open mouth gesture—is visible. When visual consonant identification was compared across initial (C[=consonant]V[=vowel]), medial (VCV), and final (VC) position (Van Son et al., 1994), identification of final consonants was 44% correct in contrast to 28% for consonants elsewhere. The point is that both optical and acoustic phonetic attributes instantiate speech features on the basis of diverse sensory information; so the visibility of speech features or phonemes cannot be inferred accurately from a simple one-to-one mapping between the visibility of speech production anatomy (e.g., lips, mouth, tongue, glottis) and speech features (e.g., voicing, place, manner, nasality).

At the same time, the reduction in visual vs. auditory speech information needs to be taken into account. The concept of the *viseme* was invented to describe and account for the somewhat stable patterns of lipreaders' phoneme confusions (Woodward and Barber, 1960; Fisher, 1968; Owens and Blazek, 1985). Visemes are sets such as /p, b, m/ that are typically formed using some grouping principle such as hierarchical clustering of consonant confusions from phoneme identification paradigms (Walden et al., 1977; Auer and Bernstein, 1997; Iverson et al., 1998). A typical rule is on the order of grouping together phonemes whose mutual confusions account for around 70% of responses. Massaro suggested that, “Because of the data-limited property of visible speech in comparison to audible speech, many phonemes are virtually indistinguishable by sight, even from a natural face, and so are expected to be easily confused” (p. 316); and that, “a difference between visemes is significant, informative, and categorical to the perceiver; a difference within a viseme class is not” (Massaro et al., 2012, p. 316).

However, most research that has used the viseme concept has involved phoneme identification tasks, for which there is a need to account for identification errors. A difference within a viseme class could be significant and informative. It could also be categorical at the level of a feature. Indeed, when presented with pairs of spoken words that differed only in terms of phonemes from within putative viseme sets, participants (deaf and normal-hearing adults) were able to identify which of the spoken words corresponded to an orthographic target word (Bernstein, 2012). That is, each word pair in the target identification paradigm was constructed so that in sequential order each of its phonemes was selected from within the same viseme. The visemes were defined along the standard lines of constructing viseme sets. An additional set of word pairs was constructed from within sets that comprised even higher levels of confusability than used to construct visemes (referred to as “phoneme equivalence classes”; Auer and Bernstein, 1997). Normal-hearing lipreaders with above-average lipreading scored between 65 and 80% correct word identification with stimuli comprising the *sub-visemic* phoneme sets (i.e., the sets of very similar phonemes). Deaf participants scored between 80 and 100% correct on those word-pairs. This would not have been possible if the phonemes that comprise visemes were not significant or informative. Thus, while there is no doubt that visual speech stimuli afford reduced phonetic detail in support of phoneme categories, there is also evidence that perceivers are not limited to perceiving viseme categories.

Interestingly, not only are perceivers able to perceive speech stimuli based on fine visual phonetic distinctions, they are also able to make judgments of the reliability of their own perceptions, apparently in terms of perceived phoneme or feature stimulus-to-response discrepancies. In a study of sentence lipreading (Demorest and Bernstein, 1997), deaf and normal-hearing adults were presented with isolated spoken sentences for open set identification of the words in the sentences. Participants were asked to type what they thought the talker had said and also to rate their confidence in their typed responses, and they received no feedback on their performance. Confidence ratings ranged from 0 = “no confidence—I guessed” to 7 = “complete confidence—I understood every word.” Scoring for how well sentences were lipread included a measure of the perceptual distance based on phoneme alignments between the stimulus and the response and was computed using a sequence comparison algorithm (Kruskal and Wish, 1978; Bernstein et al., 1994) that aligned stimulus and response phoneme sequences using visual perceptual phoneme dissimilarity weights. As an example, when the stimulus sentence was, “Why should I get up so early in the morning?” and the response was, “Watch what I'm doing in the morning,” casual inspection of the stimulus and response suggest that they have similar phoneme strings even when some of the words were incorrectly identified. The sequence comparator aligned the phonemes of these two sentences as follows (in Arpabet phonemic notation):


Stimulus: wA SUd A gEt ^p so Rli In Dx morn|G
Response: wa C-- - wxt Am du |G- In Dx morn|G


Perusal of the string alignment suggests that there were phoneme similarities even when whole words were incorrect. A visual distance score was computed for each stimulus-response pair based only on the distances between aligned *incorrect* phonemes (e.g., “S” vs. “C” in the example) normalized by stimulus length in phonemes. Correct phonemes did not contribute to distance scores. Correlations between stimulus-response distances and subjective confidence ratings showed that as stimulus-response distance (perceptual dissimilarity) increased, subjective confidence went down (reliable Pearson correlations of −0.511 for normal-hearing and −0.626 for deaf). These findings suggest that deaf and hearing adults have access to perceptual representations that preserve to some extent the phonetic information in the visual stimulus and thereby allow them to judge discrepancy between the stimulus and their own response. Thus, both this approach and the target identification approach described above reveal that sub-visemic speech information is significant and informative.

If lipreading relies on visual image processing, there should be direct relationships between the structure of the visual images and perception. A study (Jiang et al., 2007) addressed the relationship between optical recordings and visual speech perception. Recordings were made of 3-dimensional movement of the face and simultaneous video while talkers produced many different CV syllables (i.e., all the initial English consonants, followed by one of three different vowels, and spoken by four different talkers). If visual stimuli drive visual speech perception, than there should be a second-order isomorphism (Shepard and Chipman, 1970) between optical data and perception such that the dissimilarity of physical speech signals should map onto perceptual dissimilarity. The study showed that a linearly warped physical stimulus dissimilarity space was highly effective in accounting for the perceptual structure of phoneme identification for spoken CVs. Across talkers, the 3-dimensional face movement data accounted for between 46 and 66% of the variance in perceptual dissimilarities among CV stimuli.

### Spoken words

Visual spoken word recognition has been studied in experiments that were designed to investigate the pattern of visual confusions among spoken words. These studies show that visual dissimilarities affect perception to the level of spoken word identification.

For example, Mattys et al. (2002) presented isolated mono- and disyllabic spoken word stimuli to normal-hearing and deaf lipreaders for open-set visual identification. The words were selected so that they varied in terms of the number of words in the lexicon with which each was potentially confusable based on visual phoneme confusability (Iverson et al., 1998). The results showed that visual phoneme confusability predicted the relative accuracy levels for word identification by both participant groups, and phoneme errors tended to be from within groups of visually more confusable phonemes.

Auer (2002) visually presented isolated spoken monosyllabic words to deaf and normal-hearing lipreaders and modeled perception using auditory vs. visual phoneme confusion data. The visual confusions were better predictors of visual spoken word recognition than auditory confusions. Strand and Sommers (2011) followed up and tested monosyllabic words in visual-only and auditory-only (with noise background) conditions. They modeled lexical competition effects separately for visual vs. auditory phoneme similarity and showed that measures of similarity (i.e., lexical competition) that were based on one modality were not good predictors of word identification accuracy for the other modality.

### Prosody

Prosody comprises stress and intonation (Risberg and Lubker, 1978; Jesse and McQueen, 2014). Several studies have investigated visual prosody perception in normal-hearing adults (Fisher, 1969; Lansing and McConkie, 1999; Scarborough et al., 2007; Jesse and McQueen, 2014). Results suggest that prosody is perceived visually.

For example, emphatic stress for specific words such as, “We OWE you a yoyo,” vs., “We owe YOU a yoyo,” was perceived quite accurately (70%, chance = 33.3%), while perception of whether those sentences were spoken as statements or questions was perceived somewhat less accurately (60%, chance = 50%) (Bernstein et al., 1989; see also, Lansing and McConkie, 1999). Lexical stress in bisyllabic words such as *SUBject* (the noun) and *subJECT* (the verb) can be visually discriminated (62%, chance = 50%), as can phrasal stress that distinguishes (in sentences with stress on one of the names in “So, [name1] gave/sang [name2] a song from/by [name3]”) (54% correct, chance = 25%) (Scarborough et al., 2007). In the latter study, larger and faster face movements were associated with the perception of stress. For example, lower lip opening peak velocity and the size of lip opening were related to lexical stress perception.

Even whole head movement has been shown to be correlated with prosody (63% of variance accounted for between voice pitch and six components of head movement) (Munhall et al., 2004), with head movement contributing to the accuracy of speech perception in noise. Visible head movement can be used by talkers for perceiving emphasis (Lansing and McConkie, 1999).

Visual prosody perception has been studied in infants. Prosody is used in parsing connected speech and may thereby assist infants in acquiring their native language (Johnson et al., 2014). Visible prosody is likely a contributor to infants' demonstrated sensitivity to language differences in visual speech stimuli (Weikum et al., 2007).

### Interim summary

In answer to our question, What levels of speech can be perceived visually? we conclude that all levels of speech patterns (from features to connected speech) that can be heard can also be visually perceived, at least by the more skilled of lipreaders. Visual phoneme categories have internal perceptual structure that is different from that of auditory phoneme categories. At least in the better lipreaders, there may be visual modality-specific syllable or word pattern representations. Research on visual prosody suggests that it can be perceived in multisyllabic words and in connected speech. Thus, the perceptual evidence is fully compatible with the possibility that the visual speech perception relies on extensive visual modality-specific neural representations.

## An auditory representation of visual speech?

The earliest human neuroimaging studies on lipreading revealed activity in the region of primary auditory cortex, leading to discussions about the role of the auditory pathway in processing visual speech, perhaps as early as the primary auditory cortex (Sams et al., 1991; Calvert et al., 1997). Interpretations of the observed activity pointed to a role for the auditory pathway akin to its role in processing auditory speech stimuli: For example, “results show that visual information from articulatory movements has an entry into the auditory cortex” (Sams et al., 1991); “activation of primary auditory cortex during lipreading suggests that these visual cues may influence the perception of heard speech before speech sounds are categorized in auditory association cortex into distinct phonemes” (Calvert et al., 1997); “Visual speech has access to auditory sensory memory” (Möttönen et al., 2002); and “seen speech with normal time-varying characteristics appears to have preferential access to ‘purely’ auditory processing regions specialized for language” (Calvert and Campbell, 2003).

These statements were not accompanied by an explicit model or theory about how visual speech stimuli are represented by visual cortical areas upstream of auditory cortex. One reading of these statements is that rather than computing the patterns of visual speech *qua* speech within the visual system, there is a special route for visual speech to the auditory pathway where it is represented as though it were an auditory speech stimulus.

Alternatively, visual speech patterns are integrated somehow within the visual system and then projected to the primary auditory cortex where they are re-represented. However, the re-representation of information is considered to be a computationally untenable solution for the brain (von der Malsburg, 1995).

Another possibility is that visual stimuli are analyzed by the visual system only to the level of features such as motion or edges that are not integrated specifically as speech, and those feature representations are projected to the auditory pathway. But then it would be necessary to explain at what point the unbound information specific to speech was recognized as speech and was prioritized for entry into the auditory pathway. This possibility clearly suggests a “chicken and egg” problem.

Whatever its implications, there have been various attempts to confirm with neuroimaging in the human that primary auditory cortex activation levels increase following visual speech stimuli, with mixed results (Ludman et al., 2000; Bernstein et al., 2002; Calvert and Campbell, 2003; Besle et al., 2004; Pekkola et al., 2005; Okada et al., 2013). However, were visual speech prioritized for entry to auditory cortex, we might expect to see its effects more consistently.

Even when obtained, higher activation levels measured in the region of primary auditory cortex are of course not unambiguous with regard to the underlying neural response. They could for example be due to auditory imagery (Hickok et al., 2003). Or visual motion could drive the response (Okada et al., 2013). The location of primary auditory cortex could be inaccurately identified, particularly with group averaging, as non-invasive methods are imprecise in delineating the auditory core vs. belt cortex (Desai et al., 2005). Finally, a definite possibility is that activity measured with functional imaging in the region of the auditory cortex is attributable to feedback rather than visual stimulus pattern representation (Calvert et al., 2000; Schroeder et al., 2008).

There are relevant monkey data concerning the representation of input across modalities. Direct connections have been demonstrated from auditory core and parabelt to V1 in monkeys (Falchier et al., 2002) and from V2 to caudal auditory cortex (Falchier et al., 2010). These studies did not show connections from V1 to A1. The character of the connections is that of feedback through the dorsal visual pathway, commensurate with the function of representing extra-personal peripheral space and motion. “These results suggest a model in which putative unisensory visual and auditory cortices do not interact in a classical feedforward–feedback relationship but rather by way of a feedback loop. A possible implication of this organization is that the dominant effects of these connections between early sensory areas are modulatory” (Falchier et al., 2010). Importantly, monkey work has also shown that visual stimuli can modulate auditory responses in primary and secondary auditory fields *independent of the visual stimulus categories* (Kayser et al., 2008), and similar findings have been generalized to modulation of auditory cortices by somatosensory stimuli (Lemus et al., 2010). Thus, while there are functional connections, these connections between early sensory areas may serve primarily downstream modulatory functions and not upstream representation of perceptual detail needed for recognizing stimulus categories.

Overall, replication of primary auditory cortex activation by visual speech has not been completely successful, explanations invoking phonetic processing have been vague with regard to upstream visual input computations, and animal research has not been supportive of the possibility that visual speech perception is the result of representing the visual speech information through activation of auditory speech representations. The research on auditory speech processing, to which we now turn, also discourages notions about the representation of visual speech by the auditory pathway.

## The auditory representation of speech

The research on auditory speech processing is fairly clear in establishing that phonetic and phonemic speech representations in superior temporal regions beyond auditory core are viewed as modal, that is, abstracted from low-level acoustic characteristics but preserving some of their attributes. These modality specific auditory representations are not predicted to also respond to visual speech stimulus phonetic features or phonemes. Thus, our neuroanatomical model in Figure 1 posits distinct visual and auditory pathways to the level of pSTS.

Emerging work in the human suggests that neurons in the left superior temporal gyrus (STG) show selectivity to spectrotemporal acoustic cues that map to distinct phonetic features (e.g., manner of articulation) and not to distinct phonemes. Sensitivity to different phonetic features has been demonstrated in the middle and posterior STG using data-mining algorithms to identify patterns of activity in functional magnetic resonance imaging (fMRI) (Formisano et al., 2008; Kilian-Hutten et al., 2011; Humphries et al., 2013) and in intracranial (Chang et al., 2010; Steinschneider et al., 2011; Chan et al., 2014; Mesgarani et al., 2014) responses. There is now also conclusive evidence that an area in the left middle and ventral portion of STG and adjacent superior temporal sulcus (mSTG/S) is specifically sensitive to highly-familiar, over-learned, speech categories, responding more strongly to native vowels and syllables relative to spectrotemporally matched non-speech sounds (Liebenthal et al., 2005; Joanisse et al., 2007; Obleser et al., 2007; Leaver and Rauschecker, 2010; Turkeltaub and Coslett, 2010; DeWitt and Rauschecker, 2012), or relative to non-native speech sounds (Jacquemot et al., 2003; Golestani and Zatorre, 2004). Importantly, there appears to be spatial segregation within the left STG, such that dorsal STG areas largely surrounding the auditory core demonstrate sensitivity to acoustic features relevant to phonetic perception (whether embedded within speech or non-speech sounds), and a comparatively small ventral STG area adjoining the upper bank of the middle superior temporal sulcus (mSTG/S) demonstrates specificity to phonemic processing (Humphries et al., 2013). Thus, there is evidence for hierarchical organization of a ventral stream of processing in the left superior temporal cortex for the representation of phonemic information based on acoustic phonetic features.

These findings indicate at least two levels of processing for auditory phonemic information in the left lateral STG, generally consistent with the hierarchical processing of spectral and temporal sound structure during auditory object perception in belt and parabelt areas in the monkey (Rauschecker, 1998; Kaas and Hackett, 2000; Rauschecker and Tian, 2000; Rauschecker and Scott, 2009). In the monkey, selectivity for communication calls has been shown in the lateral belt (Rauschecker et al., 1995) and especially in the anterolateral area feeding into the ventral stream (Tian et al., 2001), already one synaptic level from the core, although it is possible that increased selectivity occurs along the ventral-stream hierarchy. In the human, it appears that selectivity for phoneme processing in the left mSTG/S is at least two synaptic levels downstream from the auditory core. An important implication of the foregoing findings for our discussion here is that neural representations of auditory speech features in the left STG are *modal* (and not a-modal or symbolic), as they preserve a form of the acoustic signal that is abstracted from low-level acoustic characteristics coded in hierarchically earlier auditory cortex. This intermediate level of sensory information representation (preserving the form of complex sensory features or patterns) is predicted by a computational model of categorical auditory speech perception (Harnad, 1987). The findings are also consistent with models of speech perception based primarily on acoustic features (Stevens and Wickesberg, 2002). An open question however, is how to correctly characterize neural representations in the phonemic left mSTG/S area. The anatomical proximity of this area to auditory cortex and strong specificity for speech perception over other language functions (Liebenthal et al., 2014) may suggest retention of some acoustic form (though greatly abstracted) even at this higher level of the speech processing hierarchy. Activation in areas more anterior in the STG (relative to mSTG/S) has been associated with the processing of linguistic and paralinguistic features available in larger chunks of speech such as words and sentences, for example syntax, prosody, and voice (Belin et al., 2000; Zatorre et al., 2004; Humphries et al., 2005, 2006; Hoekert et al., 2008; DeWitt and Rauschecker, 2012), whereas activation in the more ventral middle temporal cortex is associated with speech comprehension (Binder, 2000; Binder et al., 2000; Scott et al., 2000; Davis and Johnsrude, 2003; Humphries et al., 2005; DeWitt and Rauschecker, 2012).

Other areas outside the left mSTG/S have also been implicated in the neural representation of auditory phonemic information, particularly during phonological processing (i.e., when phonemic perception involves phonological awareness and phonological working memory, for example during explicit phonemic category judgment). The areas implicated in phonological processing are primarily those associated with the auditory dorsal pathway, including the posterior superior temporal gyrus (pSTG), inferior parietal cortex and ventral aspect of the precentral gyrus (Wise et al., 2001; Davis and Johnsrude, 2003; Buchsbaum et al., 2005; Hickok and Poeppel, 2007; Rauschecker and Scott, 2009; Liebenthal et al., 2010, 2013). Neurons in the supramarginal gyrus (SMG) (Caplan et al., 1997; Celsis et al., 1999; Jacquemot et al., 2003; Guenther et al., 2006; Raizada and Poldrack, 2007; Desai et al., 2008; Tourville et al., 2008) and ventral precentral gyrus (Wilson and Iacoboni, 2006; Meister et al., 2007; Chang et al., 2010; Osnes et al., 2011; Chevillet et al., 2013) may represent the somatosensory and motor properties of speech sounds, and these areas are thought to exert modulatory influences on phonemic processing. In the inferior frontal cortex (pars opercularis in particular), sensitivity to phoneme categories (Myers et al., 2009; Lee et al., 2012; Niziolek and Guenther, 2013) may be related to the role of more anterior inferior frontal cortex areas (pars orbitalis, pars triangularis) in response selection during auditory and phoneme categorization tasks.

The evidence reviewed here is consistent with the idea that both ventral and dorsal auditory streams contribute to phonemic perception. Phonemic perception in the left ventral auditory stream is organized hierarchically from dorsal STG areas surrounding the auditory core and representing acoustic phonetic features to ventral mSTG/S areas representing phoneme categories. In the dorsal auditory pathway, phonemic perception is a result of the interaction of neurons in the left pSTG representing acoustic phonetic features of speech and neurons in inferior parietal and frontal regions representing somatosensory and motor properties of speech. With respect to visual speech, the strategic location of pSTG at the junction with inferior parietal and ventral motor cortex and the multifunctionality of this area (Liebenthal et al., 2014) make it ideally suited to interact with visual speech areas and mediate the effects of visual speech input on auditory phonemic perception, an observation that has been extensively explored in the audiovisual speech processing literature, which we discuss below. However, visual speech may also exert its influence through interaction with frontal cortices, also discussed below.

### Interim summary

Research on auditory speech is producing a detailed understanding of the organization of auditory speech representations. Although far from complete, the present view is that auditory speech is processed hierarchically from basic acoustic feature representations, to phonetic features and phonemes, and then to higher-levels such as words. The evidence is strong that neural representations of auditory speech features in the left STG are modal (and not a-modal or symbolic), as they preserve an acoustic form of the signal that is abstracted from low-level acoustic characteristics coded in hierarchically earlier auditory cortex. This evidence has at least one very strong implication for visual speech perception: Visual speech is not expected to share representations with auditory speech at its early modal levels of representation.

## Multisensory speech processing research: its relevance to understanding visual speech representations

Evidence is abundant that the brain is remarkably multisensory (Foxe and Schroeder, 2005; Schroeder and Foxe, 2005; Ghazanfar and Schroeder, 2006; Kayser et al., 2012), in the sense that it affords diverse neural mechanisms for integration and/or interaction (Stein et al., 2010) among different sensory inputs. Research on audiovisual speech processing has focused on discovering those mechanisms. But the approaches have mostly not been designed to answer questions about the organization of unisensory speech representations: It has focused on answering questions such as whether there are influences from visual speech in classically defined auditory cortical areas (e.g., Sams et al., 1991; Calvert et al., 1997, 1999; Bernstein et al., 2002; Pekkola et al., 2005), whether relative information clarity in auditory vs. visual stimuli affects neural network activations (Nath and Beauchamp, 2011; Stevenson et al., 2012), and whether audiovisual integration demonstrates the principle of inverse effectiveness [(Stein and Meredith, 1993) i.e., multisensory gain is inversely related to unisensory stimulus effectiveness] (e.g., Calvert, 2001; Beauchamp, 2005; Stevenson et al., 2012). Studies of multisensensory speech interactions commonly depend on designs that use audiovisual, auditory-only, and visual-only speech stimuli without controls designed to test hypotheses about the detailed organization of unisensory processing. Unisensory stimuli are used in the research as controls and for defining multisensory sites. For example, a common control for visual-only speech is a still frame of the talker or a no-stimulus baseline (e.g., Sekiyama et al., 2003; Stevenson and James, 2009; Nath and Beauchamp, 2011, 2012; Barros-Loscertales et al., 2013; Okada et al., 2013).

Because of the interest in multisensory interactions, research has focused on putative integration sites such as the pSTS (Calvert et al., 2000; Wright et al., 2003; Callan et al., 2004; Nath and Beauchamp, 2012; Stevenson et al., 2012), which is part of both the auditory and visual pathways (see Figure 1). The left pSTS is routinely activated during audiovisual phoneme perception (e.g., Calvert, 2001; Sekiyama et al., 2003; Miller and D'Esposito, 2005; Stevenson and James, 2009; Nath and Beauchamp, 2011). However, high-resolution examination of pSTS demonstrates clusters of neurons in the dorsal and ventral bank of bilateral pSTS that respond to either auditory or visual input, with intervening clusters responding most strongly to audiovisual input (Beauchamp et al., 2004). What speech pattern attributes may be coded by such multisensory vs. unisensory clusters has not to our knowledge been investigated. In monkey, the STS has been found to have stronger feedback, as well as feed forward, connections with auditory and visual association rather than core areas (Seltzer and Pandya, 1994; Lewis and Van Essen, 2000; Foxe et al., 2002; Ghazanfar et al., 2005; Smiley et al., 2007).

### Interim summary

To this point, we have reviewed the evidence that demonstrates visual perception of every psycholinguistic level of speech stimuli. We have discussed the hypothesis that visual speech might be represented through the auditory speech pathway. But our review of the auditory speech pathways suggests that representations are considered to be modal to the level of phonetic and phonemic speech representations in superior temporal regions beyond auditory core. Our view of the audiovisual speech processing literature is that its focus on multisensory interactions has resulted in limited evidence about the organization of the unisensory speech pathways. However, the expectation from the study of pSTS is that visual speech representations are projected to pSTS, and the question then is what information is represented through the visual system.

## Organization of the bottom-up visual pathways and implications for speech representations

Since the 1980s, the visual system organization has been described in terms of a *ventral* stream associated with form and object perception, and a *dorsal* stream associated with movement, space perception, and visually guided actions (Ungerleider and Mishkin, 1982; Goodale et al., 1994; Ungerleider and Haxby, 1994; Logothetis and Sheinberg, 1996; Zeki, 2005). Both streams effect hierarchical organization with each level of representations building on preceding ones, and higher levels are more invariant to surface characteristics of visual objects, such as orientation and size. But perception is not limited to higher level representations. That is, perceivers have access to multiple levels of the pathways (Hochstein and Ahissar, 2002; Zeki, 2005).

In its general outline, the visual ventral stream extends from V1 in the occipital lobe to V2, V3, and V4, and into ventral temporal cortex and frontal cortex. The dorsal stream extends from V1 into V2, V3, V5/MT, and dorsal temporal areas including STS, extending further to parietal and frontal areas. This organization has long been known to be not strictly hierarchical and to comprise cross-talk among areas (Felleman and Van Essen, 1991; for a recent review, Perry and Fallah, 2014). A recent proposal for a three-stream model (Weiner and Grill-Spector, 2013) implicates communication between ventral and dorsal streams for language processing, to which we return below.

### Visual pathway organizations of faces, orthography, and sign language perception

The organization of visual speech pathways could possibly be in common with the organization of other types of input, including faces, orthography, and possibly sign language that share certain attributes with visual speech. Face processing obviously must to be considered in relationship to visual speech (Campbell et al., 1986; Campbell, 2011). Faces and visual speech are usually co-present, and faces are a rich source of many types of socially significant information (Allison et al., 2000; Haxby et al., 2002)—such as person identity, emotion, affect, and gaze. The “core face processing network” is generally considered to include the right lateral portion of the fusiform gyrus (FG) referred to as the fusiform face area (FFA), the lateral surface of the inferior occipital gyrus referred to as the occipital face area (OFA), and an area of the pSTS (Kanwisher et al., 1997; Fox et al., 2009). There is ample evidence that face and body representations are distinct (Downing et al., 2006; Weiner and Grill-Spector, 2013), and that body and visual speech representations are distinct (Santi et al., 2003). Face areas in cortex may be localized more reliably with moving than with still face stimuli (Fox et al., 2009). In a comparison between static and dynamic non-speech face images, right FFA and OFA did not prefer dynamic images but right posterior and anterior STS did (Pitcher et al., 2011). However, in a study with different frame rates and scrambled vs. ordered frames of non-speech facial motion stimuli, differential effects were observed in face processing areas (Schultz et al., 2013): Bilaterally, STS was more responsive to dynamic and ordered frames, but FFA and OFA were not sensitive to the order of frames, only to the amount of image diversity in the scrambled frames.

Visual speech activations have also been recorded in the FG (Calvert and Campbell, 2003; Capek et al., 2008), leading to the suggestion that visual speech processing uses the FFA (Campbell, 2011). However, as noted above, the moving face is likely to more effectively activate face representations in the FFA, and diverse static images activate FFA more effectively than a single image. An independent face localizer is needed to functionally define the FFA region of interest (ROI) (Kanwisher et al., 1997), because it cannot be defined based on anatomy alone. But FFA localizers have not typically been used with visual speech. To determine whether FFA represents speech distinctions such as speech features or phonemes also requires methods that are sensitive to differences across speech features or phonemes within FFA ROIs. Below, we discuss results when an independent FFA localizer was used, and FFA was shown responsive to speech stimuli but less so than to non-speech face movements (Bernstein et al., 2011).

Although orthography is visually different from visual speech, both stimulus types likely make contact with higher-level mechanisms of spoken language; and both may involve recognizing words through fairly automatized whole-word recognition and also phonological analyses. Dorsal and ventral pathways have been shown to represent orthographic stimuli (Pugh et al., 2000; Jobard et al., 2003; Borowsky et al., 2006). With respect to language, as with the auditory ventral pathway, the visual ventral pathway organized from occipital through inferior temporal to frontal regions is characterized as having responsibility for relating orthographic forms to word meanings. The ventral stream could be viewed as representing specifically the forms of familiar words and exception words (e.g., letter strings with atypical spelling-to-sound correspondences, e.g., “pint”), and mapping them to word pronunciations.

We are not suggesting that lipreading is built on reading. If anything, the opposite would be more likely, given that speech is encountered earlier in development, and given that orthography is an evolutionarily recent form of visual input. But the dual stream organization observed in reading research could be related to the processing resources needed by lipreaders, inasmuch as a more skilled lipreader would be expected to have more automatized access to certain lexical items as well as need for phonological processing; and a less skilled lipreader might have greater reliance on dorsal stream processing to glean fragmentary phonetic or phonemic category information and construct possible lexical items in stimuli. Spoken words with few or no visually similar competitors (Auer and Bernstein, 1997; Iverson et al., 1998) might be particularly good candidates for skilled lipreading via whole-word representations. Likewise, the wide individual differences among lipreaders (Bernstein et al., 2000; Auer and Bernstein, 2007) could be the consequence of differential development of visual speech pathways.

Sign language perception is also visually distinct from visual speech but might have some commonality with lipreading. Classical language areas (inferior frontal and posterior temporal areas) within the left hemisphere were recruited by American Sign Language in deaf and hearing native signers (Bavelier et al., 1998). However, lipreading, auditory speech perception, and reading are united by their basis in spoken language (MacSweeney et al., 2008). In addition, deaf users of sign language likely have experienced extensive neuroplastic changes in cortical and sub-cortical organization (MacSweeney et al., 2004; Fine et al., 2005; Auer et al., 2007; Kral and Eggermont, 2007; Lyness et al., 2014) such that there could be commonality in the visual pathway for representing the configurations and dynamics of visual speech and signs. Both types of stimuli are reliant on form and motion. But research on sign language processing emphasizes commonalities at higher psycholinguistic levels (MacSweeney et al., 2002). However, consistent with reading, there is some evidence for dual-stream processing of sign language. Hearing native signers activated left inferior termporal gyrus (ITG) and STS more with British sign language than with Tic Tac, a manual system used by bookmakers at race tracks (MacSweeney et al., 2004) in contrast with hearing non-signers. Hearing native signers more than non-native signers activated ITG and middle temporal gyrus (MTG) for word lists vs. a still baseline, supporting a general role for the ventral pathway in fluent word recognition regardless of the form of the stimuli (speech, sign, orthography).

### Organization of visual speech processing

In our model of auditory and visual modality-specific processing (Figure 1), we assume the standard visual pathways labeled “dorsal” and “ventral,” because we expect that visual speech is subject to visual system organization. But the pathway labeled “dorsal” may actually correspond to the lateral pathway in Weiner and Grill-Spector (2013), which we discuss further below. The model is highly schematized, because in fact there are few results in the literature that speak directly to how the levels of speech that can be perceived by vision are neurally represented.

The literature on visual speech processing is fairly consistent in showing bilateral posterior activation in areas associated with ventral and dorsal visual pathways (Calvert et al., 1997; Campbell et al., 2001; Nishitani and Hari, 2002; Skipper et al., 2005; Bernstein et al., 2008a, 2011; Capek et al., 2008; Murase et al., 2008; Okada and Hickok, 2009; Ponton et al., 2009; Files et al., 2013). When spoken digits were contrasted with gurning (Campbell et al., 2001), bilateral FG, and right STG and MTG were more activated by speech; left IT areas were more active in the contrast between speech and a still face. When still images of speech gestures were contrasted against the baseline of a still face, bilateral FG, occipito-temporal junction, MTG, and left STS were activated (Calvert and Campbell, 2003); and dynamic stimuli were more effective than still speech in those same areas, except the bilateral lingual gyri. In a study in which spoken words were contrasted with a still face image (Capek et al., 2008), widespread bilateral activation was reported in ventral and lateral temporal areas. In a magnetoencephalography study (Nishitani and Hari, 2002), still speech images evoked a progression of activation from occipital to lateral temporal cortex labeled as pSTS. In a study in which short sentences were contrasted with videos of gurning and also with static faces (Hall et al., 2005), there was extensive bilateral but greater left-hemisphere activation in ventral and lateral middle temporal cortices. MTG activation extended to the pSTS. When lipreading syllables and gurning were contrasted (Okada and Hickok, 2009), left posterior MTG/STS, and STG activation was obtained. When participants were imaged with positron emission tomography (PET) (Paulesu et al., 2003) while watching a still face, a face saying words, and the backwards video of the same words (backwards and forwards speech contains segments that are not different, such as vowels and transitions into and out of consonants), activations were obtained bilaterally in STG, bilateral superior temporal cortex and V5/MT. Connected speech in a story was presented in a lipreading condition that did not require any attempt to understand the story (Skipper et al., 2005), however significant activity was restricted to occipital gyri and right ITG. This result seems difficult to interpret in light of the possibility that participants were not paying attention to the speech information.

Several generalizations can be made about the above studies. A variety of stimuli was contrasted mostly against a fixed image or gurning. For the most part, visual speech stimuli reliably activated areas that can be identified within the classical ventral and dorsal visual streams. Activity was typically widespread. Activations were often bilateral although not in strictly homologous locations. Typically, results were reported as group averages and smoothed activations. Cortical surface renderings of individual activations on native anatomy were not presented. So the published results are not very helpful with regard to individual differences in anatomical location or extent of activation. Independent functional localizers for visual areas such as the FFA and V5/MT were not used, although activations generally consistent with their locations were discussed. As a group, these studies provide confirmation that the ventral and dorsal visual pathways can be activated by visual speech, but they were not designed to investigate in any detail how visual speech is represented through the pathways. To do so would have required using various controls for low-level features and higher-level objects such as faces, taking into account factors such as sensitivity to movement in FFA, using contrasts reflective of the organization of speech such as between different phonemes or speech levels, and taking into account individual variations in visual speech perception.

Bernstein et al. (2011) sought to begin to address several of the previous limitations in methodology that limit ability to determine the organization of visual speech representations in high-level vision. They used functional localizers, a variety of speech, non-speech, and moving control stimuli, and contrasted video vs. point-light images. Participants underwent independent localizer scans for the FFA, the lateral occipital complex (LOC) associated with image structure (Grill-Spector et al., 2001), and the V5/MT motion processing areas. The experimental stimuli were nonsense syllables that were selected for their visual dissimilarity [“du,” “sha,” “zi,” “fa,” “ta,” “bi,” “wi,” “dhu” (i.e., the voiced “th”), “ku,” “li,” and “mu”]. In separate conditions, a variety of non-speech face gestures (“puff,” “kiss,” “raspberry,” “growl,” “yawn,” “smirk,” “fishface,” “chew,” “gurn,” “nose wiggle,” and “frown-to-smile”) was presented. A parallel set of stimuli and controls was created based on 3-dimensional optical recordings that were made simultaneously with the video recordings. The optical recordings were of the motion of retro-reflectors positioned at 17 locations with most positions around the mouth, jaw, and cheeks. The optical recordings were used to generate point-light videos (Johansson, 1973). The point-light stimuli presented speech and non-speech motion patterns without other natural visual features such as the talker's eye gaze, shape of face components (mouth, etc.) and general appearance. Speech and non-speech stimuli were easy to discern in the point-light displays. The point-light stimulus patterns were hypothesized to represent the structure of the speech information in motion and to some extent also configuration in terms of the arrangement of the dots and shape from motion (Johansson, 1973). Point-light speech stimuli enhance the intelligibility of acoustic speech in noise (Rosenblum et al., 1996) and can interfere with audiovisual speech perception when they are incongruent (Rosenblum and Saldana, 1996). Visual controls were created from the speech and non-speech stimuli by dividing the area of the mouth and jaw into 100 square tiles. The order of frames within each tile was scrambled across sequential temporal groups of three frames. Using this scheme, the stimulus energy/luminance of the original stimuli was maintained. The control stimuli had the appearance of a face with square patches of unrelated movement.

The results showed that *non-speech* face gestures significantly activated the FFA, LOC, and V5/MT ROIs more strongly than *speech* face-gestures, supporting the expectation that none of those visual areas are selective for speech patterns. Detailed analysis of the motion data from the optical image recordings suggested that the reduced activity to speech in FFA, LOC, and V5/MT ROIs was not due to different speed of motion across stimulus types. One surprise, given its ubiquity in the literature, was that the gurn stimulus had much higher motion speed than the speech or the other non-speech stimuli. However, removal of the results that were obtained when gurns were presented did not change the overall pattern of results in ROIs.

The main experimental results were used to search for areas selective for speech independent of media (that is across point-light and video stimuli). Because point-light stimuli present primarily motion information with very much reduced configural information and no face detail, activations in conjunctions were interpreted as areas most concerned with speech patterns. Although there were activations in the right temporal cortices, the left-hemisphere activations were viewed as candidates for visual speech representations in high-level vision areas feeding forward into left-lateralized language areas. Based on individual and group results, contiguous areas of posterior MTG and STS were shown to be selective for speech. The localized posterior temporal speech selective area was dubbed the temporal visual speech area (TVSA). Figure 1 shows the approximate location of TVSA, with the caveat that precise locations varied with individual anatomy (see Supplementary Figure 7, Bernstein et al., 2011, for individual ROIs). On an individual-participant basis, the speech activations in pSTS/pMTG were more anterior than adjacent cortex that preferred non-speech gestures. They demonstrated preliminary evidence for a positive correlation with individual lipreading scores. The finding of a visual speech area (i.e., TVSA) posterior and inferior to pSTS is consistent with the idea that TVSA is a modal area in high-level vision, possibly distinct from multisensory pSTS.

In order to examine sensitivity to phonemic speech dissimilarity in the putative TVSA, Files et al. (2013) used a visual mismatch negativity (vMMN) paradigm to present consonant-vowel stimuli. The vMMN is elicited by change in the regularity of a sequence of visual stimuli (Pazo-Alvarez et al., 2003; Winkler and Czigler, 2012). Visual speech stimuli were selected to be *near* (ambiguous yet phonemically discriminable) or *far* (clearly different phonemes) in physical and speech perceptual distance based on a quantitative model of visual speech dissimilarity (Jiang et al., 2007). The hypothesis was tested that the left posterior temporal cortex (i.e., TVSA) has tuning for visual speech, but the right homologous cortex has tuning for discriminable speech stimuli regardless of whether they can be labeled reliably as different phonemes. Discrimination among speech stimuli that are phonemically ambiguous would be expected of cortical areas that process non-speech face movements that can vary continuously (Puce et al., 2000, 2003; Miki et al., 2004; Thompson et al., 2007; Bernstein et al., 2011) such as with different extent of mouth opening or with different motion velocities. The prediction was that regardless of perceptual distance the right hemisphere would generate the vMMN across discriminable stimuli; but only *far* phonemic contrasts would generate the vMMN on the left. Larger, more discriminable phoneme differences would be expected to feed forward to the left-lateralized language cortex.

Several attempts had previously been made to obtain vMMNs for visual speech category differences (Sams et al., 1991; Colin et al., 2002, 2004; Saint-Amour et al., 2007; Ponton et al., 2009; Winkler and Czigler, 2012). In those studies, either the vMMN was not obtained, the mismatch response was at a very long latency suggesting that it was not related to input pattern processing *per se*, or the obtained vMMN could be attributed to non-speech visual stimulus attributes. In Files et al. (2013), the stimulus selection was designed to defend against mismatch responses due to stimulus differences other than phoneme membership (be it perceptually near or far). Two tokens were presented for each phoneme category so that the vMMN would not be attributable to individual stimulus token differences. Stimuli were shifted spatially from trial to trial to defend against low-level stimulus change such as slight head or eye position variation on the screen. Care was taken to identify the temporal points in each stimulus at which the moving speech images deviated from each other, and those points were used to measure the vMMN latencies.

Current density reconstructions (Fuchs et al., 1999) and statistical analyses using clusters of posterior temporal electrodes showed reliable left-hemisphere responses to individual stimuli and vMMNs to *far* stimulus phonemic category change. On the right, vMMNs were obtained with both *far* and *near* changes. Responses were in the range of latencies observed with non-speech face gestures stimuli. Current density reconstructions demonstrated consistent patterns of posterior temporal responses in the region of pMTG to the visual speech stimuli (Figures 4–6 in Files et al., 2013), with the caveat that reconstructions are limited in their spatial resolution. The finding of hemispheric differences in the pattern of vMMN responses, with greater sensitivity to smaller difference on the right, was interpreted as evidence the left posterior temporal cortex (putative TVSA) processes phonemic patterns that feed forward into language processing areas, and that more analog processing is carried out on the right as would be required for perceiving non-categorical, non-speech face gestures.

### Proposed model

Figure 1 proposes a schematic model of the auditory and visual pathways and interactions between them. The primary prediction of the model is that modal representations of visual speech exist to the level of the TVSA, and that this area is posterior and ventral to the multisensory pSTS. We acknowledge that far too little experimental evidence currently exists to determine with any precision what the organization of visual speech representations is through the visual system.

Lipreading must rely on processing of both configural features and/or stimulus patterns, and dynamic stimulus features. Although the processing of configural features is typically associated with the ventral visual stream and that of dynamic features with the dorsal visual stream, both types of information may be represented along both ventral and dorsal streams to some extent. Form has long been known to be perceived from motion (Johansson, 1973). Current research on interactions between dorsal and ventral stream processing in object and motion perception (for a review see Perry and Fallah, 2014) supports the view that object segmentation and representation is assisted by motion features, and motion representations are affected by object form input. Perry and Fallah propose that these interactions may occur further downstream from the visual motion area (MT). The conjunction results in Bernstein et al. (2011) using point-light and video speech stimuli that localized TVSA in pMTG seems consistent with the suggestion that TVSA is responsive to both form and motion. Observations of speech activations in IT could be due to configural processing but likely are supported by motion processing, given cross-talk between ventral and dorsal streams.

It is an entirely open question whether the identified TVSA has an internal organization that could support processing in both the dorsal and ventral visual streams, for example, as an anterior area that is part of the ventral stream and a posterior area that is part of the dorsal stream, similar to the anterior-to-posterior differentiation in the left STG for auditory speech perception. It also remains an open question whether TVSA overlaps at least partially with other high-level visual areas, for example LOC in the ventral visual stream. We suggest that such questions can be answered only with careful mapping of the different functional areas within individuals and taking into account perceptual variability.

Recently, a three-stream model was proposed by Weiner and Grill-Spector (2013). In their model, the visual system is organized in terms of a dorsal vision-action stream, a ventral visual perception stream for recognition of forms such as objects and faces, and a lateral stream concerned with form, visual dynamics and language, among other functions. The lateral pathway comprises the lateral occipital sulcus, the middle occipital gyrus, the posterior inferior temporal sulcus, and the MTG extending into V5/MT. The lateral stream communicates with both the parietal cortex of the dorsal stream and the inferior temporal cortex of the ventral stream. This arrangement is compatible with what is known to date about visual speech processing. Weiner and Grill-Spector do not elaborate on the possible role of their proposed lateral stream, but research on visual speech processing could contribute to a better understanding of this proposed lateral pathway.

### The role of frontal and parietal areas in visual speech perception

Our discussion of a neural model of visual speech perception has focused thus far on high-level vision areas. However, as for auditory speech perception, other motor and somatosensory areas in the frontal and parietal cortex have also been implicated in visual speech perception, particularly within the theoretical framework that posits a human frontal cortex mirror neuron system (Rizzolatti and Arbib, 1998). This view is compatible with the longstanding motor theory of speech perception (Liberman and Mattingly, 1985) and with the evidence for modulatory effects of the somatomotor system on auditory phonemic perception reviewed above (Wilson et al., 2004; Meister et al., 2007; Möttönen and Watkins, 2009; Osnes et al., 2011) in the context of a somatomotor role for both the auditory and visual dorsal streams (Rauschecker and Scott, 2009).

Frontal cortex activation is commonly observed with audiovisual or visual speech perception (e.g., MacSweeney et al., 2000; Bernstein et al., 2002, 2011; Möttönen et al., 2002; Callan et al., 2003; Calvert and Campbell, 2003; Paulesu et al., 2003; Sekiyama et al., 2003; Miller and D'Esposito, 2005; Ojanen et al., 2005; Skipper et al., 2005, 2007b; Okada and Hickok, 2009; Matchin et al., 2014). Inferior frontal activations during overt categorization of speech stimuli have been attributed to a role of this area in cognitive control and domain-general category computation (Hasson et al., 2007; Myers et al., 2009). Somatomotor system engagement is often observed in the context of failure to integrate audiovisual stimuli. Because visual speech is typically less intelligible than acoustic speech, or is presented in the context of noisy acoustic speech, speech somatomotor activity observed during audiovisual speech perception could arise due to conflict resolution with degraded speech (Miller and D'Esposito, 2005; Callan et al., 2014) or due to response biases (Venezia et al., 2012). However, unlike auditory and visual cortices, the frontal cortex does not appear to play a critical role in the perception of clear speech, that is, in the accurate representation of stimulus patterns.

A study (Hasson et al., 2007) comparing rapid adaptation (Grill-Spector and Malach, 2001) effects with veridical vs. perceptual speech stimulus repetition concluded that areas in inferior frontal gyrus (IFG) coded for perceptual rather than sensory physical stimulus properties. Thus, when a mismatched visual “ka” and auditory “pa” were preceded by an audiovisual “ta”—the syllable typically heard with the mismatched stimuli—adaptation in IFG was similar to that with a veridical audiovisual “ta.” Thus, the observed adaptation effects followed perceived category change and not sensory stimulus change.

Callan et al. (2014) presented CVC English words under audiovisual conditions with three levels of noise, auditory-only conditions with three levels of noise, visual-only speech, and a still face baseline. The task was forced-choice identification of the vowel. Visual-only and audiovisual stimuli activated left IFG and ventral premotor cortex. Visual-only activation was greater than audiovisual in a dorsal part of the premotor cortex, implying some modal effects even in frontal cortex. However, there was not an examination of categorization effects within the dorsal premotor cortex, so it is not at all clear what the modality-specific response is attributable to.

The SMG has also been a focus in research on audiovisual speech integration (Hasson et al., 2007; Bernstein et al., 2008a,b; Arnal et al., 2009; Dick et al., 2010). Activation in this area has been observed with visual-only speech (Chu et al., 2013) and with auditory speech (Caplan et al., 1997; Celsis et al., 1999; Jacquemot et al., 2003; Guenther et al., 2006; Raizada and Poldrack, 2007; Desai et al., 2008; Tourville et al., 2008; Liebenthal et al., 2013). Left SMG is sensitive to individual differences in processing incongruity of visual speech (Hasson et al., 2007). It is sensitive to the degree of stimulus incongruity measured independently across auditory and visual speech, which suggests also that some modal aspect of representation extends to the SMG (Bernstein et al., 2008b).

Overall, common activation in parietal and frontal areas in response to auditory and visual speech is expected (see Figure 1), in light of the evidence that such areas participate in higher-level (amodal) aspects of language processing.

## Summary and conclusions

Our inquiry into the visual speech perception literature shows that all levels of speech patterns that can be heard can also be seen, with the proviso that perception is subject to large individual differences. The perceptual evidence is highly valuable, because it leads to a strong rationale for undertaking research to discover how the brain represents visual speech.

We discussed the implication from neuroimaging results that visual speech has special status in possibly being represented not by the visual system but by the auditory system. Our review of the literature, including the organization of the auditory pathways leads us to doubt the validity of that suggestion. Modal representations of auditory speech exist beyond the auditory core areas that have been observed to respond to visual speech. We are in accord with the view that those activations are related to feedback, modulatory effects (Calvert et al., 1999) and not to the representation of visual speech patterns *per se*.

Neuroimaging literature on lipreading shows widespread and diverse activity in the classical ventral and dorsal visual pathways in response to visual speech. However, the literature has for the most part not addressed in sufficient detail the organization and specificity of visual pathways for visual speech perception. A main drawback has been the use of baseline stimuli such as a still face or gurns to contrast with visual speech. Our recent fMRI and EEG studies with more in-depth focus on visual speech attributes provide evidence for a left posterior temporal area, TVSA, in high-level vision, possibly the recipient of both ventral and dorsal stream input, and sensitive to phonetic and phonemic speech attributes.

While there is not at the moment sufficient evidence for making detailed neuroanatomical predictions regarding the organization of the visual cortex for visual speech processing, we make the following empirically testable predictions: (1) The visual perception of speech relies on visual pathway representations of speech *qua* speech. That is, visual speech perception relies on stimulus patterns represented through visual pathways. (2) A proposed site of these, the TVSA, has been demonstrated in posterior temporal cortex, ventral and posterior to multisensory posterior superior temporal sulcus (pSTS). TVSA may feed modal information to downstream multisensory integration sites in pSTS. (3) Given that visual speech has dynamic and configural features that together are important for visual speech perception, neural representation of visual speech in feed forward visual pathways are expected to integrate to some extent across these features, possibly at the level of TVSA. Thus, a rigid division of the visual system into a dorsal and a ventral stream likely is not an adequate description for visual speech. Rather, the expectation is that there is cross-talk between areas in these paths for the processing of visual speech. (4) Visual speech information is expected to be fed forward from the occipital cortex to both the inferior parietal cortex along a dorsal visual pathway, and to the middle temporal cortex along a ventral visual pathway. Given the implication of the occipital-parietal (dorsal) visual stream in visual control of motor actions and spatial short-term memory (amongst other functions), we expect that the neural representations of visual speech in high-level areas of this stream may maintain more of the veridical, dynamic, and sequential information of the visual input, similar to neural representations of speech in the dorsal auditory stream (Wise et al., 2001; Buchsbaum et al., 2005; Hickok and Poeppel, 2007; Rauschecker and Scott, 2009; Liebenthal et al., 2010). Given the implication of the occipito-temporal (ventral) visual stream in visual object recognition and long-term memory, we expect that neural representations in high-level areas of this stream may be highly abstracted from the visual input, similar to the neural representations of speech phonemes in the ventral auditory pathway (Liebenthal et al., 2005; Joanisse et al., 2007; Obleser et al., 2007; Leaver and Rauschecker, 2010; Turkeltaub and Coslett, 2010; DeWitt and Rauschecker, 2012).

We make the following suggestions for future research: (1) Given individual differences in perception and functional location of TVSA, detailed examination is needed within individuals to understand the organization of visual speech representations; (2) To understand fully how neural processes underlying visual and auditory speech perception interact, examination is needed, again within individuals, of the organization of both visual and auditory pathways for speech perception. (3) The ability to visually perceive all the psycholinguistic levels of speech calls for research both within and across psycholinguistic levels (i.e., phonetic features, phonemes, syllables, words, and prosody) of organization. In principle, the organization of visual speech processing cannot be determined based only on unspecific contrasts such as speech stimuli vs. still face images.

### Conflict of interest statement

The authors declare that the research was conducted in the absence of any commercial or financial relationships that could be construed as a potential conflict of interest.

## Footnotes

^1^The term *lipreading* is used in this paper to refer to perceiving speech by vision. An alternate term that appears in the literature is *speechreading*. This term is sometimes used to emphasize the point that visual speech perception is more than perception of lips, and sometimes it is used to refer to visual speech perception augmented by residual hearing in individuals with hearing impairments.
